# A Biomechanical Evaluation of a Novel Interspinous Process Device: In Vitro Flexibility Assessment and Finite Element Analysis

**DOI:** 10.3390/bioengineering12040384

**Published:** 2025-04-03

**Authors:** Hangkai Shen, Chuanguang Ju, Tao Gao, Jia Zhu, Weiqiang Liu

**Affiliations:** 1 China United Engineering Corporation, First Industrial Design and Research Institute, Hangzhou 310000, China; 2 Biomechanics & Biotechnology Lab, Research Institute of Tsinghua University in Shenzhen, Shenzhen 518000, China; 3 Yantai Affiliated Hospital, Binzhou Med University, Yantai 264100, China

**Keywords:** cadaveric experiment, finite element, lumbar biomechanics, interspinous process device

## Abstract

The interspinous process device (IPD) has emerged as a viable alternative for managing lumbar degenerative pathologies. Nevertheless, limited research exists regarding mechanical failure modes including device failure and spinous process fracture. This study developed a novel IPD (IPD-NEW) and systematically evaluated its biomechanical characteristics through finite element (FE) analysis and in vitro cadaveric biomechanical testing. Six human L1–L5 lumbar specimens were subjected to mechanical testing under four experimental conditions: (1) Intact spine (control); (2) L3–L4 implanted with IPD-NEW; (3) L3–L4 implanted with Wallis device; (4) L3–L4 implanted with Coflex device. Segmental range of motion (ROM) was quantified across all test conditions. A validated L1–L5 finite element model was subsequently employed to assess biomechanical responses under both static and vertical vibration loading regimes. Comparative analysis revealed that IPD-NEW demonstrated comparable segmental ROM to the Wallis device while exhibiting lower rigidity than the Coflex implant. The novel design effectively preserved physiological spinal mobility while enhancing load distribution capacity. IPD-NEW demonstrated notable reductions in facet joint forces, device stress concentrations, and spinous process loading compared to conventional implants, particularly under vibrational loading conditions. These findings suggest that IPD-NEW may mitigate risks associated with facetogenic pain, device failure, and spinous process fracture through optimized load redistribution.

## 1. Introduction

Conventional lumbar interbody fusion (LIF) remains the gold-standard surgical intervention for lumbar spinal stenosis (LSS) treatment [1]. Although LIF demonstrates satisfactory clinical efficacy [2], emerging evidence suggests it may induce biomechanical alterations in adjacent spinal segments, including elevated segmental motion and stress concentrations [3,4]. These biomechanical changes constitute a recognized risk factor for the development of adjacent segment degeneration (ASD) during long-term follow-up. Clinical studies report substantial heterogeneity in post-LIF radiographic ASD incidence, with estimates ranging from 5.2% to 100% across patient cohorts [5].

Interspinous process devices (IPDs) have been developed as motion-preserving alternatives to lumbar interbody fusion (LIF) for reducing adjacent segment degeneration (ASD) risk in lumbar spinal stenosis (LSS) treatment. These devices utilize dynamic stabilization to preserve physiological motion while optimizing load distribution across spinal segments [6]. Clinical studies confirm IPDs’ effectiveness, demonstrating both positive clinical outcomes and radiographic improvements [7,8]. Systematic reviews and meta-analyses comparing IPDs with LIF [9,10] reveal equivalent postoperative recovery, alongside shorter surgical duration, reduced hospitalization time, and remarkably lower ASD rates.

The positive clinical outcomes of IPDs have prompted extensive investigations into their biomechanical performance. For example, Wilke et al. [11] demonstrated in cadaveric studies that IPDs remarkably reduce intradiscal pressure (IDP) and range of motion (ROM) during extension, with minimal effects on axial rotation and lateral bending. Shen et al. [12] employed the finite element (FE) method to analyze the biomechanics of various IPDs. Additionally, Yin et al. [13] investigated the biomechanical behavior of IPDs under vibrational loads and found that combining IPDs with interbody cages effectively absorbs vibrational energy at adjacent segments. Several studies have also used optimized topology methods to improve the structural design of IPDs [6,14].

While IPDs effectively reduce the occurrence of ASD, their use is associated with an increased risk of revision surgeries [10]. Device failure [15] and spinous process fractures [16] constitute primary indications requiring surgical revision. In clinical practice, there are also concerns about their capacity to provide long-term stability and preserve spinal alignment. A critical consideration in the use of IPDs is their impact on segmental lordosis and adjacent segment mechanics. Recent studies emphasize the importance of segmental lordosis and pelvic parameters, such as pelvic tilt, in determining outcomes following lumbar fusion procedures. For instance, a recent review highlights the relationship between segmental lordosis, pelvic alignment, and the development of ASD [17]. These findings emphasize the need for careful patient selection and precise surgical planning when considering IPDs, especially in the context of their potential to induce segmental distraction and kyphosis. However, the biomechanical mechanisms underlying IPD-related device failure and spinous process fractures remain poorly understood.

Therefore, this study was designed to develop a novel IPD (IPD-NEW) to mitigate device failure and spinous process fracture risks while evaluating its biomechanical performance. A three-dimensional FE model of the L1–L5 lumbar spine was developed to conduct comparative biomechanical evaluations between IPD-NEW and the existing Wallis/Coflex systems. The analysis incorporated both static and vibrational loading conditions. In addition, we investigated the biomechanical stability provided by IPD-NEW in an in vitro flexibility experiment by using six L1–L5 human cadaveric lumbar spines.

## 2. Methods and Materials

### 2.1. FE Models of the L1–L5 Lumbar Spine

**Figure 1:**
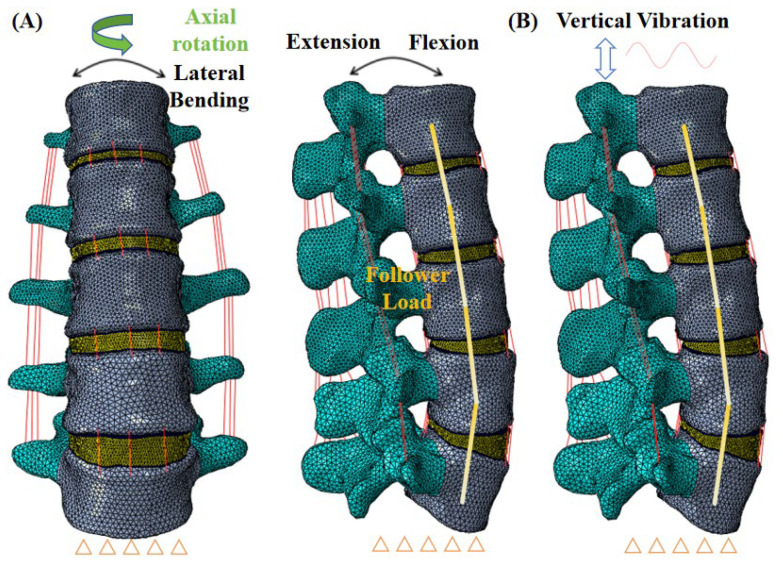
Finite element simulation of the L1–L5 spinal segment, and the lower surface of the L5 segment is fixed: (**A**) under static loading; (**B**) under vertical vibration.

In the current study, we utilized a previously established and verified FE model of the human lumbar spine (L1–L5) [12], as illustrated in Figure 1A,B. The spinal structure was reconstructed using computed tomography (CT) scans, which were 0.7 mm in slice thickness, taken from a healthy female volunteer (aged 37, 158 cm, and 52 kg). A cumulative total of 492 CT scans were converted into a three-dimensional geometric model and meshed using the Hypermesh 14.0 software. Our model encompassed elements such as cancellous bone, cortical bone, posterior elements, intervertebral discs, cartilaginous endplates, and all seven types of ligaments. The cortical bone thickness was designated at 1 mm [18], whereas the cartilaginous endplate was set at 0.5 mm [18]. The intervertebral disc was composed of the annulus fibrosus and the nucleus pulposus [18]. Ligamentous structures were represented with truss elements that only resist tension [19]. Interactions at the facet joints were modeled as frictionless surface-to-surface contacts [19,20]. The comprehensive FE model comprised 123,468 nodes and 578,066 elements, and simulations were executed using the Abaqus 6.14 software.

To affirm the accuracy of the L1–L5 spinal model, we adhered to the validation procedures established in prior research [21]. The lower surface of L5 was immobilized in all the planes, and distinct moments were exerted at the L1 level—specifically, 8 Nm for flexion, 6 Nm for extension, 4 Nm for axial rotation, and 6 Nm for lateral bending. Subsequently, our simulation outcomes were compared with Renner et al.’s experimental data [21]. Moreover, under escalating compressive loads ranging from 100 to 400 N, we contrasted the axial displacement and IDP at the L4–L5 segment with the experimental results reported by previous research [22,23].

**Figure 2:**
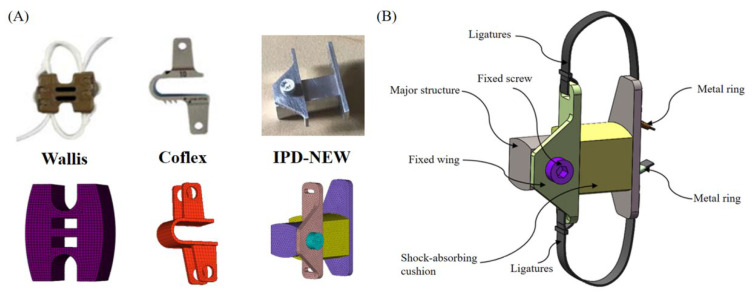
Interspinous process devices compared in the current research: (**A**) Wallis, Coflex, and IPD-NEW; (**B**) the structure of IPD-NEW.

**Figure 3:**
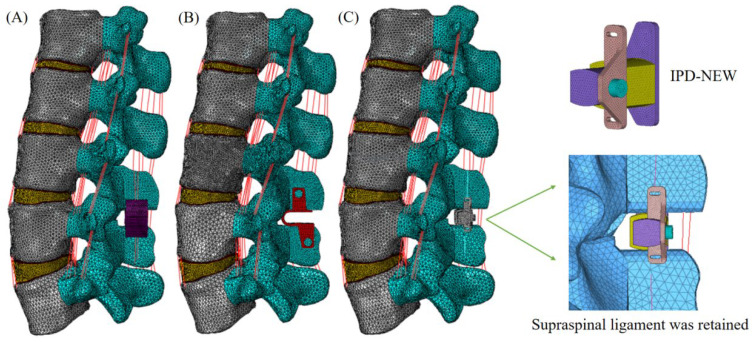
FE models of various interspinous process devices: (**A**) L3–L4 Wallis; (**B**) L3–L4 Coflex; (**C**) L3–L4 IPD-NEW.

The surgical FE models were created utilizing the verified intact lumbar model as a foundation. At the L3–L4 level (as shown in Figure 2A), three distinct IPDs—Wallis, Coflex, and IPD-NEW—were inserted. The Wallis was fabricated from polyether ether ketone (PEEK), while the Coflex was constructed using a titanium alloy. The structure of IPD-NEW is shown in Figure 2B, and its main material was titanium alloy. IPD-NEW also included a shock-absorbing cushion, of which the main material was medical thermoplastic polyurethane (TPU). For the procedure of inserting the IPDs, a portion of the L3–L4 spinous process was excised to create flush contact surfaces as documented in [12,24]. In the cases of the Wallis and IPD-NEW, the interspinous ligament was removed prior to the implantation; the bone-to-IPD contact was then modeled using a frictional surface-to-surface interaction (friction coefficient of 0.2), as referenced in [25]. With the Coflex device, both the interspinous and supraspinous ligaments were resected; the device’s teeth were modeled with a higher friction coefficient of 0.8, while other contact areas had a reduced coefficient of 0.1, both values cited from [25]. The surgical models are depicted in Figure 3. Furthermore, the material properties of each component are detailed in Table 1.

The base of the L5 vertebra was secured from all movements. A 280 N follower force, aligning with the lumbar spine’s curvature, was imposed to mimic the influence of body weight [18]. Subsequently, two distinct loading scenarios were investigated. The initial scenario involved a static load where a torque of 7.5 Nm was applied to the upper surface of L1 to replicate motions of flexion, extension, lateral bending, and axial rotation [18]. Simulations were conducted to assess the ROM, facet joint force (FJF), stresses in the device, and the stress on the spinous processes. The second scenario involved dynamic loading, simulating whole-body vibration (WBV) by applying an oscillatory vertical load (±40 N, 5 Hz, 2 s) on the upper surface of L1 [19,20]. Then, the dynamic results of FJF, device stress, and spinous process stress were researched.

### 2.2. In Vitro Testing

In the in vitro study, six cadaveric lumbar columns of L1–L5 were utilized. These specimens were sourced from the Research Institute of Tsinghua University in Shenzhen, and the research was carried out following the institutional medical ethics procedure. The specimens included 3 males and 3 females, aged 39–78 years (60.8 ± 13.6). All the lumbar spine specimens were ensured with no absence of fractures and deformities by radiographs. Bone mineral density of the lumbar spine was measured using quantitative computed tomography (126.8 ± 24.9 mg/cm^3^), indicating no presence of osteoporosis. Paravertebral musculature was carefully resected, and the spinal ligaments, joints, and intervertebral discs were preserved [28].

**Figure 4:**
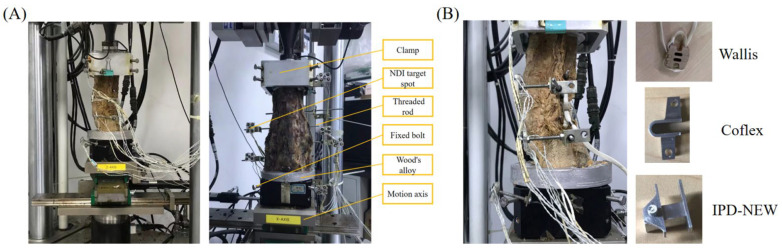
In vitro experiments with L1–L5 segments: (**A**) intact spine; (**B**) surgery group with various interspinous process devices.

The study was conducted using the MTS Bionix Spine Simulation Machine. The lower end of the lumbar spine was secured in every direction using Wood’s metal, while torque was exerted at the L1 level via a hydraulic mechanism, as depicted in Figure 4A. The testing environment was maintained at an ambient temperature of 23 °C with a humidity range of 35–80%. Periodically, 0.9% saline solution was applied to the specimens to maintain their hydration [28]. Each lumbar segment was equipped with an NDI marker comprising four target points, with the segment’s position defined by the NDI marker’s coordinates. A 280 N compressive force was aligned with the lumbar spine’s curvature, succeeded by a 7.5 Nm torque at the L1 segment to replicate motions of flexion, extension, lateral bending, and axial rotation [18], at a rate of 0.1° per second. Each motion was conducted three times, with the ROM being documented, averaged, and analyzed. Initially, specimens were assessed in their intact state, followed by tests with the Wallis, IPD-NEW, and Coflex implants at the L3–L4 level, as shown in Figure 4B.

## 3. Results

### 3.1. Validation of the Lumbar Spine FE Model

**Figure 5:**
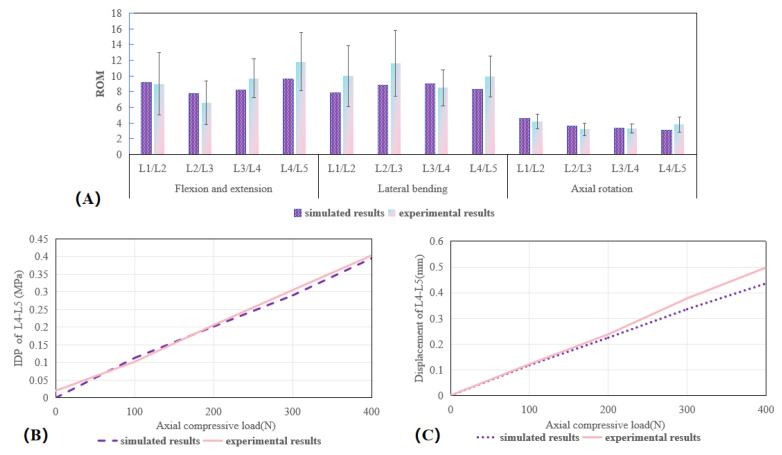
Verification of the lumbar model: (**A**) ROM for L1–L5 spinal segments; (**B**) IDP at the L4–L5 level; (**C**) axial displacement of the L4–L5 segment.

According to the established methodology, we compared our simulated ROM outcomes with those from Renner et al.’s experimental data [21]. The anticipated ROM for each segment fell within the range of one standard deviation from the biomechanical cadaver assessments reported by Renner et al. [21], as depicted in Figure 5A. Furthermore, with escalating preloads, the forecasted L4–L5 intradiscal pressure (IDP) and axial displacement were in alignment with the in vitro findings presented by Berkson et al. [22] and Brinckmann et al. [23], as shown in Figure 5B,C. Consequently, the finite element model utilized in this study was confirmed to be valid.

### 3.2. ROM (In Vitro Results)

**Figure 6:**
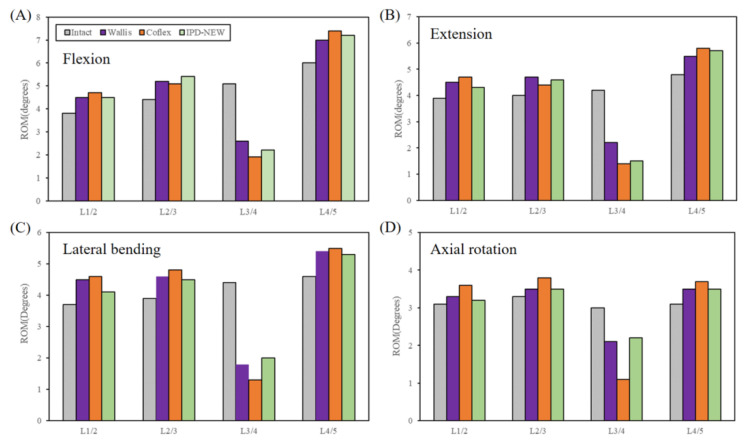
In vitro experimental results of range of motion.

Figure 6 illustrates the ROM for each segment during flexion–extension, lateral bending–axial rotation motion modes. At the L3–L4 segment, the ROM for IPD-NEW was lower than that of Wallis during flexion–extension motion, but higher during bending–rotation motion. Additionally, IPD-NEW exhibited a greater ROM than Coflex across all the motion types. In the adjacent segments, surgical cases showed an increased ROM compared to the intact cases. Moreover, no remarkable differences were found in the ROM of adjacent segments among Wallis, Coflex, and IPD-NEW.

To assess the impact of the small sample size on our findings, we conducted a sensitivity analysis using Bootstrap resampling and confidence interval estimation (Supplementary Materials Figures S1–S16). The Bootstrap resampling results indicated that the mean values of ROMs exhibited relatively narrow fluctuations, suggesting that the results of ROMs are robust despite the limited sample size.

### 3.3. Range of Motion (FE Results)

**Figure 7:**
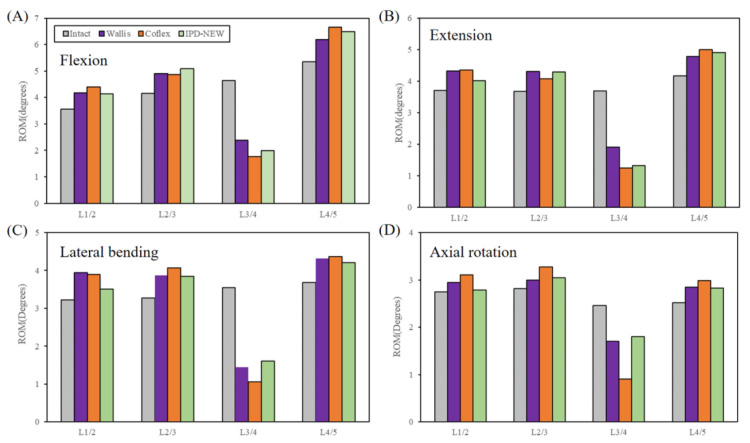
Finite element results of range of motion.

Figure 7 presents the anticipated ROM for each spinal segment. The FE outcomes were found to be 5–26% less than the in vitro data. Nevertheless, compared among IPD-NEW, Wallis, and Coflex, the trends of the simulated results were consistent with the cadaveric experiment.

### 3.4. Facet Joint Force

**Figure 8:**
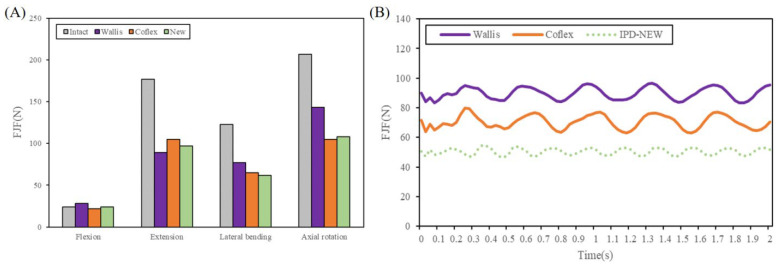
Facet joint force of each group: (**A**) under static loading conditions; (**B**) during vertical vibrations.

Under static loading conditions, Figure 8A illustrates the anticipated facet joint forces (FJF) at the L3–L4 level. The surgical models showed a notable reduction in FJF compared to the intact lumbar model, particularly during extension, axial rotation, and lateral bending. When compared to the Wallis device, IPD-NEW resulted in a 19.5% decrease in FJF during lateral bending and a 24.5% reduction in axial rotation while showing a 9.0% increase in extension. In comparison to Coflex, IPD-NEW led to a 7.6% and 4.6% decrease in FJF during extension and lateral bending, respectively, but exhibited a 2.9% increase in axial rotation.

For dynamic loading conditions, the FJF’s dynamic responses are shown in Figure 8B. Table 2 summarizes the maximum and minimum values along with the vibration amplitudes. IPD-NEW demonstrated the lowest dynamic responses of FJF during vertical vibrations when compared to Wallis and Coflex.

### 3.5. Device Stress

**Figure 9:**
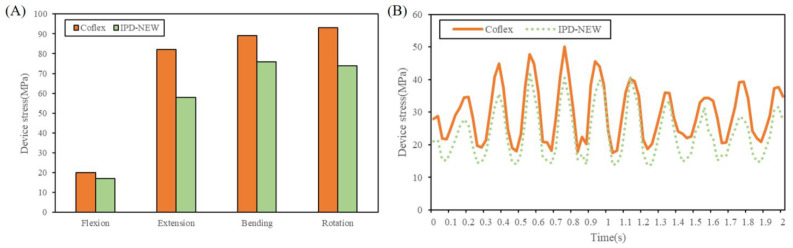
Device stress of each group: (**A**) under static loading conditions; (**B**) during vertical vibrations.

In conditions of static loads, Figure 9A compared the device stress of Coflex and IPD-NEW. Wallis was not compared because its main material (PEEK) was different from Coflex and IPD-NEW (Ti6Al4V). Compared with Coflex, IPD-NEW decreased the device stress by 15%, 29%, 15%, and 20% in flexion–extension motion and bending–rotation motion, respectively.

In conditions of vertical vibration, Figure 9B depicted the fluctuations in device stress as dynamic responses. In contrast to Coflex, IPD-NEW achieved a reduction of 15% in the peak device stress, 22% in the trough values, and 12% in the stress amplitudes.

### 3.6. Spinous Process Stress

**Figure 10:**
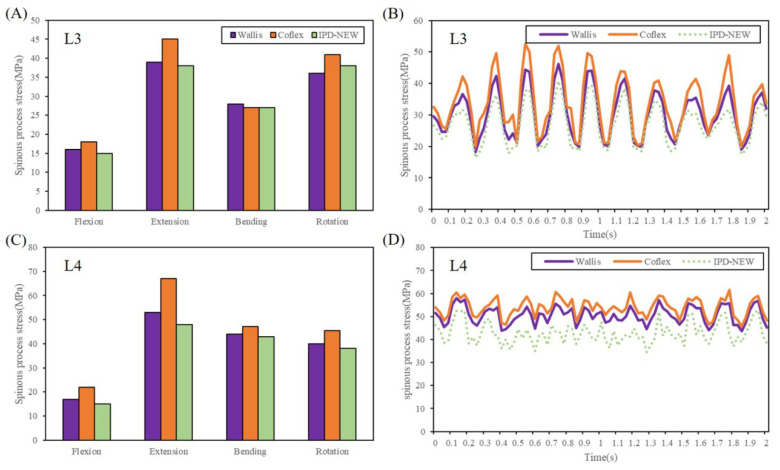
Spinous process stress of each group: (**A**) L3 spinous process stress during static loads; (**B**) L3 spinous process stress during vertical vibration; (**C**) L4 spinous process stress during static loads; (**D**) L4 spinous process stress during vertical vibration.

In conditions of static loads, Figure 10A,C showed the predicted results of spinous process stress. In contrast to both Wallis and Coflex, IPD-NEW resulted in a reduction in stress on the spinous process in all types of motion except for axial rotation. For the L3 segment, the stress on the spinous process with IPD-NEW was slightly greater than that observed with Wallis during axial rotation.

Figure 10B,D illustrate the dynamic responses of the spinous process stresses. When compared to Wallis and Coflex, IPD-NEW notably reduced the dynamic responses of spinous process stresses in the adjacent segments.

## 4. Discussion

Recently, IPD has served as an alternative therapeutic option for lumbar degenerative diseases because of its minimal invasive characteristics. The aim of the IPD is to enhance stability following the alleviation of nerve compression at the surgical level [6]. Although IPDs showed satisfactory clinical outcomes, there have also been several reports about device breaks [14] and spinous process fractures [15,16]. Therefore, this study designed a new IPD and investigated its biomechanical performance by FE methods and in vitro cadaveric experiments.

In the in vitro study of spinal flexibility, six human lumbar specimens of L1–L5 were utilized. As illustrated in Figure 6, compared among the different surgical cases, the L3–L4 ROM of IPD-NEW was higher than that of Coflex, and lower than that of Wallis in flexion–extension motion. During bending–rotation motion, the ROM at the L3–L4 segment for IPD-NEW exceeded that of both Wallis and Coflex. The IPD-NEW demonstrated comparable segmental stabilization to the Wallis system, though inferior to the Coflex device. This disparity stems from Coflex’s superior structural rigidity, which resulted in more constrained instrumented segment ROM across all the movement planes [11]. Notably, IPDs predominantly modulated ROM in flexion–extension movements, with minimal effects on lateral bending–axial rotation, consistent with Wilke et al.’s cadaveric data [11] and Shen et al.’s computational models [12]. This biomechanical behavior may originate from IPD-induced facet joint distraction mechanisms, given these joints’ critical role in controlling rotational motions [6]. Additionally, adjacent segment ROM showed no substantial inter-device variations among the three implants. Chen et al. [14] also reported a similar result in their biomechanical study: at the adjacent segments, the variations in the ROMs for each IPD were within 5%, 7%, 6%, and 12% during flexion–extension motion and bending–rotation motion, respectively. This indicates that the stiffness of the adjacent segments remained largely unchanged following the implantation of the IPD.

In addition, the biomechanical properties of lumbar spine specimens are influenced by factors such as intervertebral disc degeneration, bone mineral density, gender, and age. Disc degeneration is remarkably negatively correlated with lumbar motion. Degenerated specimens have reduced disc height, water content, and annulus fibrosus rupture, and even herniation, markedly decreasing lumbar motion [29]. Furthermore, disc degeneration may alter load distribution and increase stress on the facet joints, further restricting mobility [30]. Therefore, we ensured that all the lumbar spine specimens exhibited no degeneration by radiographs. The effect of bone mineral density on lumbar spine mobility is relatively indirect, with some studies suggesting that osteoporosis may increase vertebral fragility, thereby limiting the ROM [31,32]. In this study, the bone mineral density of all the specimens fell within the normal range (126.8 ± 24.9 mg/cm^3^), thus exerting minimal impact on the ROM measurements. Additionally, gender and age may also affect pelvic anatomical structure, disc elasticity, and ligament tension, consequently influencing mobility outcomes. The current study included specimens from 3 males and 3 females, aged from middle to old age. Future studies should incorporate a larger and more diverse study group to minimize the potential biases introduced by age and gender variations.

In addition to the in vitro test, FE analysis was also conducted to investigate the ROM, FJF, device stress, and spinous process stress of the lumbar spine. Figure 7 displays the predicted ROM of each segment after IPD implantation. Compared to the experimental results from the cadaveric specimens, the findings and trends from the FE simulations were consistent. However, the ROM predicted by the FE model was relatively lower, with a reduction of approximately 5–26%. This discrepancy may be attributed to the smaller dimensional size of the FE model relative to the actual specimen size. Studies have demonstrated that the ROM of the lumbar spine is influenced by a combination of various anatomical and biomechanical factors, including intervertebral disc height, vertebral body height, and diameter of the vertebral canal [33,34,35]. An increase in intervertebral disc height is correlated with a greater ROM, as taller discs provide a larger space for elastic deformation [33,34], allowing for enhanced compression, extension, and lateral bending. And, an increase in vertebral body height may enhance spinal flexibility, thereby expanding the ROM. In addition, a smaller vertebral canal diameter may restrict the mobility space of the lumbar structures, thereby indirectly affecting the ROM, particularly in lateral bending and axial rotation.

Previous investigations have documented that vertical oscillations markedly amplify the stress on lumbar discs [36,37,38], presenting a higher risk than those under static loading scenarios. Therefore, in addition to the static loading condition, the vibration loading condition was also simulated in the current study.

A fundamental concept of IPD is to reduce pain associated with facet joints [6,11]. In our research, under static loading, the FJF of IPD-NEW was found to be less than Wallis’s in lateral bending and axial rotation, yet moderately elevated (9.0%) in extension. In comparison to Coflex, IPD-NEW reduced the FJF during extension and lateral bending, although it showed a slight increase of 2.9% in axial rotation. In conditions of vertical vibration, we observed that Coflex lowered the dynamic responses of FJF when compared to Wallis. This outcome is consistent with the results reported by Yin et al. [13] who found that the “U structure” of Coflex could absorb some vibration energy during vertical vibration. In addition, IPD-NEW remarkably reduced the peak value, trough value, and oscillation amplitudes of FJF during vertical vibration (Table 2). This result indicated that IPD-NEW may be helpful to prevent facet joint pain, especially during vibration.

Previous biomechanical studies have reported that device breaks may be caused by the stress concentration of the implants [39]. In addition, Bae et al. [40] reported that the failure rate due to device break was approximately 3.2% in their clinical study. Figure 9A,B compared the device stress of Coflex and IPD-NEW. The results showed that IPD-NEW reduced the device stress by 15–29% during static loading conditions, and decreased the vibration amplitudes by 12% during vertical vibration. This result indicated that IPD-NEW could lower the risk of device break.

Few studies have investigated the spinous process stress after IPD implantation. In Guo et al.’s FE study [6], they reported that compared with flexion, the spinous process stress was much higher in extension and axial rotation, and a comparable pattern was identified in this research. Furthermore, the research revealed that during static loading conditions, IPD-NEW slightly decreased the spinous process stress, while during vibration loading conditions, IPD-NEW decreased the maximum values by 9.7–22.5%, and vibration amplitudes by 12.9–25.6%. Thus, from a biomechanical perspective, IPD-NEW proved beneficial in reducing the likelihood of spinous process fractures, particularly under conditions of vertical vibration.

Biomechanically, IPDs function by distracting the spinous processes, which increases the spinal canal and foraminal dimensions, potentially alleviating symptoms of neural compression. However, a key concern is their tendency to induce segmental distraction and kyphosis. Our study demonstrates that the IPD-NEW may mitigate the risks associated with facetogenic pain, device failure, and spinous process fracture through optimized load redistribution. However, clinical applications should consider patient-specific factors. IPDs may be used for mild to moderate spinal stenosis or degenerative disc disease. They should be avoided in cases of significant kyphosis or anterolisthesis [41]. In some cases, IPDs may be used in conjunction with interbody fusion to maintain or restore disk height and improve spinal alignment, and this approach provides enhanced stability to the surgical segment [42]. The decision to use IPDs with interbody fusion should be based on a comprehensive assessment of the patient’s spinal alignment, pelvic parameters, and overall clinical presentation.

The findings of this study provide biomechanical evidence for the efficacy of IPD-NEW in reducing facet joint forces, device stress, and spinous process loading. However, these results need to be validated through clinical trials, including postoperative follow-up and complication statistics, to assess the translational value of our findings. Future research should aim to investigate the long-term clinical outcomes of IPD-NEW in patients with lumbar degenerative diseases.

In addition, the sample size of six cadaveric lumbar spine specimens in the in vitro study may limit the statistical power of our findings. A sensitivity analysis was conducted to evaluate the impact of sample size on the results, which confirmed the robustness of our conclusions despite the limited number of specimens. However, it is necessary to include a larger sample size to further validate these findings.

The limitations of this study need to be acknowledged. Firstly, only six human L1–L5 lumbar specimens were employed in the in vitro flexibility experiment. In our future study, more specimens should be researched. Secondly, the average size of the lumbar specimens was larger than that of the FE model, which made the predicted value lower than the experimental value. Nevertheless, the tendencies and findings from both the in vitro test and the FE analysis were aligned. In future studies, the FE model should be developed based on the actual dimensions of the specimens to further enhance precision and reliability. In the current study, the impacts of muscle force and degenerative diseases (such as reduced disc height, spondylolisthesis, or arthritic facet joints) on biomechanical responses were not adequately considered. These factors are crucial for the stability and motor complexity of the lumbar spine [43]. In addition, osteoporosis can remarkably affect the biomechanical properties of the lumbar spine [44,45]. Therefore, the next target of our research group is to add muscle tissue to the lumbar model and investigate the impact of osteoporosis on the lumbar spine.

## 5. Conclusions

This study designed a new IPD and investigated its biomechanical performances by cadaveric experiments and the FE method. In general, IPD-NEW showed similar ROM compared with Wallis and lower rigidity compared with Coflex. IPD-NEW was capable of maintaining spinal mobility while enhancing the load distribution across spinal motion segments. In addition, the main advantage of IPD-NEW was that it decreased the facet joint force, device stress, and spinous process stress, especially during vertical vibration. IPD-NEW may be helpful in preventing facet joint pain and reducing the likelihood of device break and spinous process fracture.

## Figures and Tables

**Table 1:** Material properties of the FE models.

Components of the Model	Young’s Modulus (MPa)	Poisson Ratio	Sectional Area (mm^2^)	Density (kg/mm^3^)	References
Cortical bone	12,000	0.3		1.7 × 10^−6^	Zhang et al. [18]
Cancellous bone	100	0.2		1.1 × 10^−6^	
Posterior bone	3500	0.25		1.4 × 10^−6^	
Endplate	24	0.25		1.2 × 10^−6^	Liu et al. [26]
Nucleus pulposus	1	0.49		1.02 × 10^−6^	Zhang et al. [18]
Annulus fibrosus	4.2	0.45		1.05 × 10^−6^	
Anterior longitudinal ligament	20	0.3	63.7	1 × 10^−6^	
Posterior longitudinal ligament	20	0.3	20	1 × 10^−6^	
Ligament flava	19.5	0.3	40	1 × 10^−6^	
Interspinal ligament	11.6	0.3	40	1 × 10^−6^	
Supraspinal ligament	15	0.3	30	1 × 10^−6^	
Intertransverse ligament	58.7	0.3	3.6	1 × 10^−6^	
Capsular ligament	32.9	0.3	60	1 × 10^−6^	
Coflex (Ti6Al4V)	110,000	0.3		4.5 × 10^−6^	Guo et al. [6]
Wallis (PEEK)	3500	0.4		1.32 × 10^−6^	Park et al. [25]
IPD-NEW (Ti6Al4V)	110,000	0.3		4.5 × 10^−6^	Guo et al. [6]
Ligatures	2400	0.4		1.0 × 10^−6^	Park et al. [25]
TPU cushion	Hyperelastic, Mooney–Rivlin C01 = 17.4, C10 = −11.11, C02 = 3.134			0.8 × 10^−6^	Wang et al. [27]

**Table 2:** Summary of the dynamic results.

	Wallis			Coflex			IPD-NEW		
Dynamic Responses	Max	Min	VA	Max	Min	VA	Max	Min	VA
FJF (N)									
L3–L4 FJF	96.5	83.1	13.4	79.8	63.0	16.8	54.7	46.8	7.9
Device stress (N)									
IPD stress	-	-	-	50.1	17.5	32.6	42.4	13.7	28.7
Interspinous process stress (MPa)									
L3 stress	46.2	18.2	28.0	52.6	19.8	32.8	40.8	16.4	24.4
L4 stress	58.1	43.6	14.5	61.6	45.6	16.0	52.7	34.6	18.1

## Data Availability

Data are contained within the article.

## Supplementary Materials

The following supporting information can be downloaded at: https://www.mdpi.com/article/10.3390/bioengineering12040384/s1.
