# Supplementary material for: A Biomechanical Evaluation of a Novel Interspinous Process Device: In Vitro Flexibility Assessment and Finite Element Analysis
Source: Bioengineering (Basel). 2025 Apr 3;12(4):384. doi: 10.3390/bioengineering12040384 (PMC12024796; doi:10.3390/bioengineering12040384)
Supplement: Supplementary file 1 [file bioengineering-12-00384-s001.zip › bioengineering-3491066-supplementary.pdf]

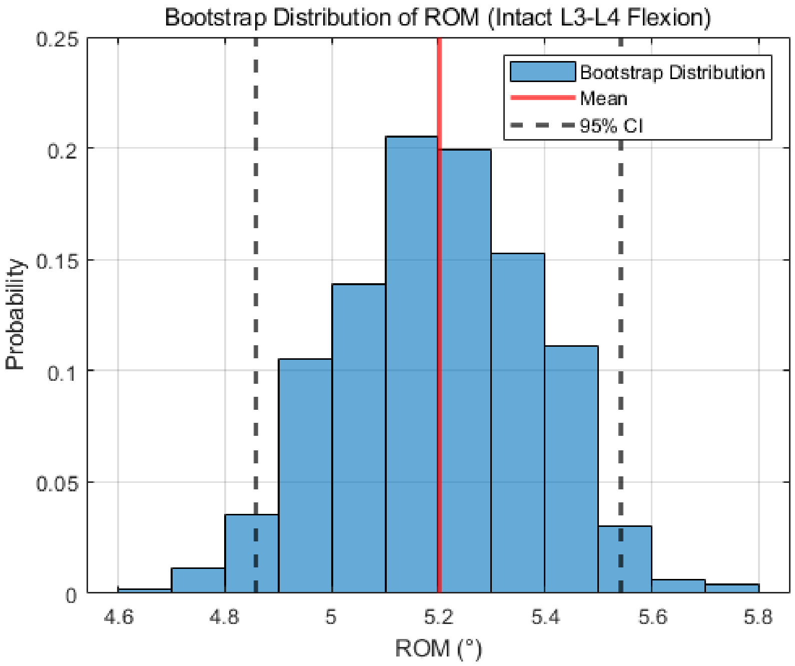

Supplementary Material Figure S1. Bootstrap Distribution of ROM (Intact L3-L4 Flexion), 95% Confidence Interval: [4.86°, 5.54°]

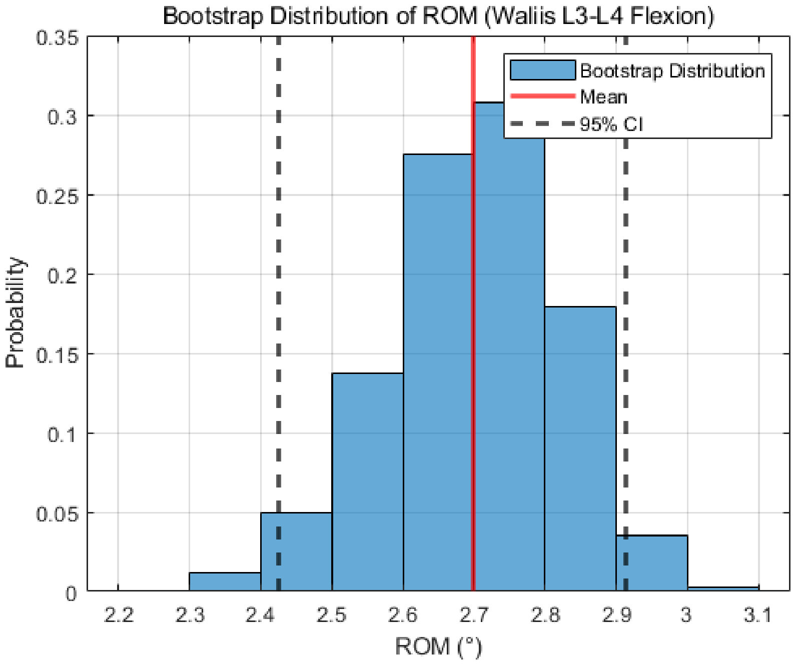

Supplementary Material Figure S2. Bootstrap Distribution of ROM (Wallis L3-L4 Flexion), 95% Confidence Interval: [2.43°, 2.91°]

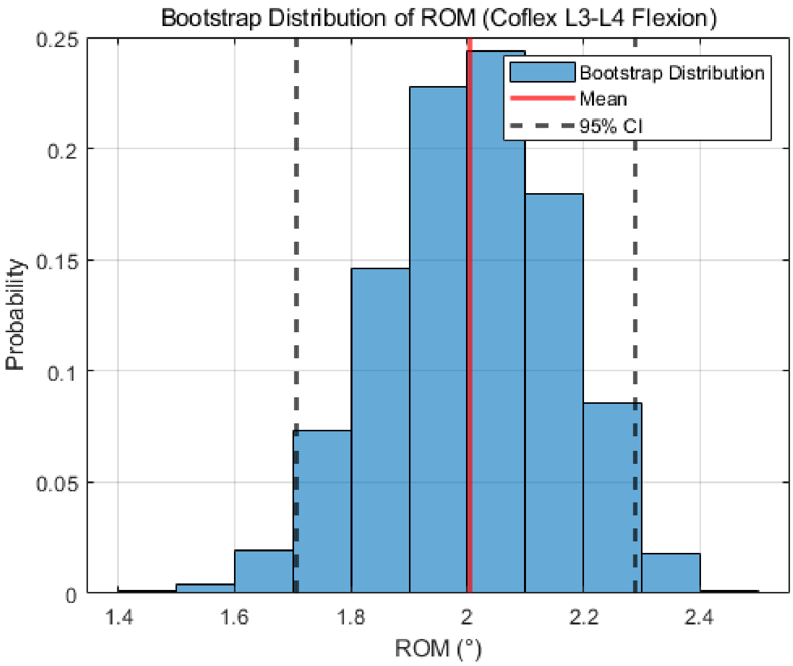

Supplementary Material Figure S3. Bootstrap Distribution of ROM (Coflex L3-L4 Flexion), 95% Confidence Interval: [1.70°, 2.29°]

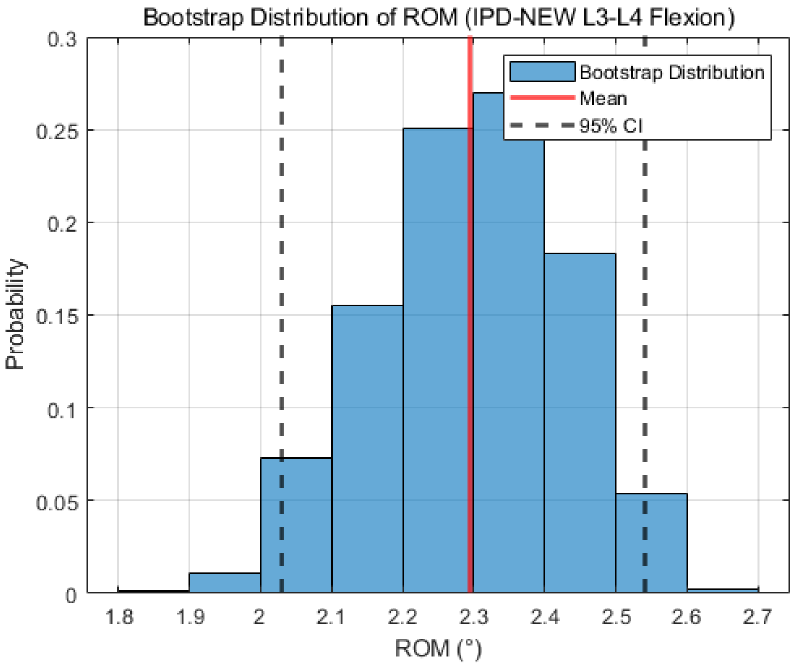

Supplementary Material Figure S4. Bootstrap Distribution of ROM (IPD-NEW L3-L4 Flexion), 95% Confidence Interval: [2.03°, 2.54°]

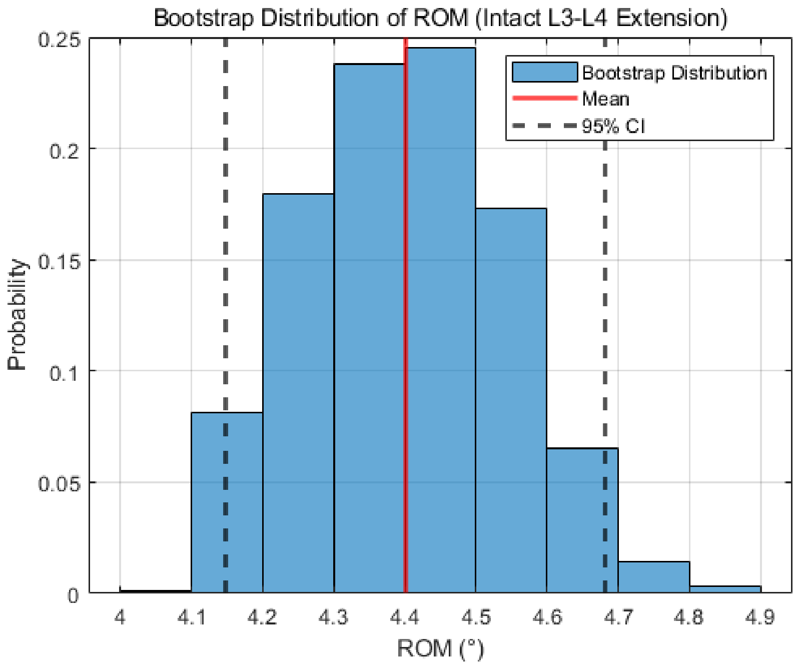

Supplementary Material Figure S5. Bootstrap Distribution of ROM (Intact L3-L4 Extension), 95% Confidence Interval: [4.15°, 4.68°]

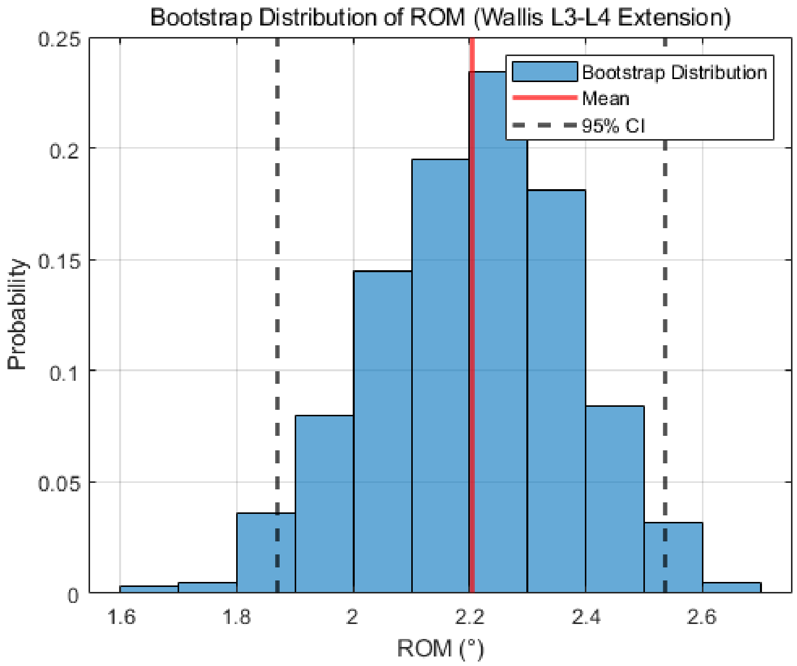

Supplementary Material Figure S6. Bootstrap Distribution of ROM (Wallis L3-L4 Extension), 95% Confidence Interval: [1.87°, 2.54°]

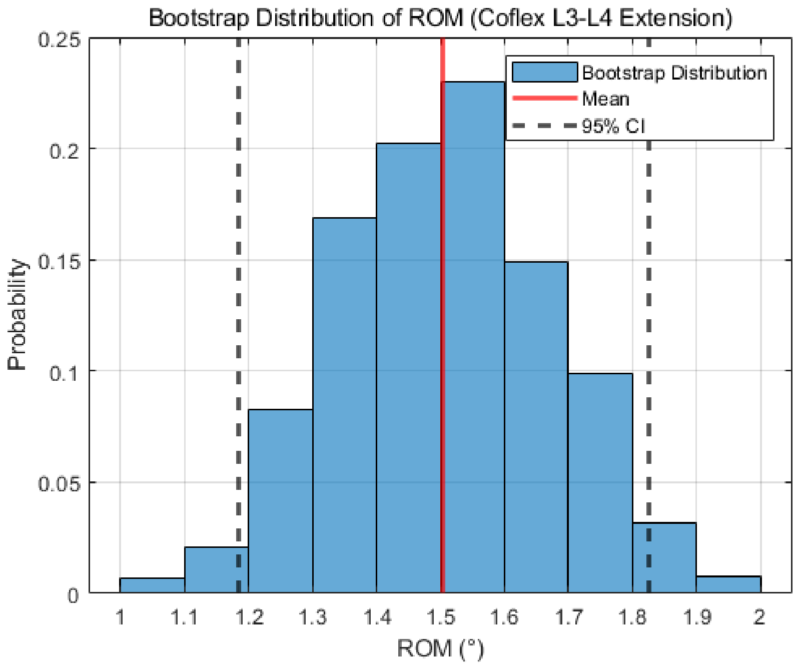

Supplementary Material Figure S7. Bootstrap Distribution of ROM (Coflex L3-L4 Extension), 95% Confidence Interval: [1.18°, 1.83°]

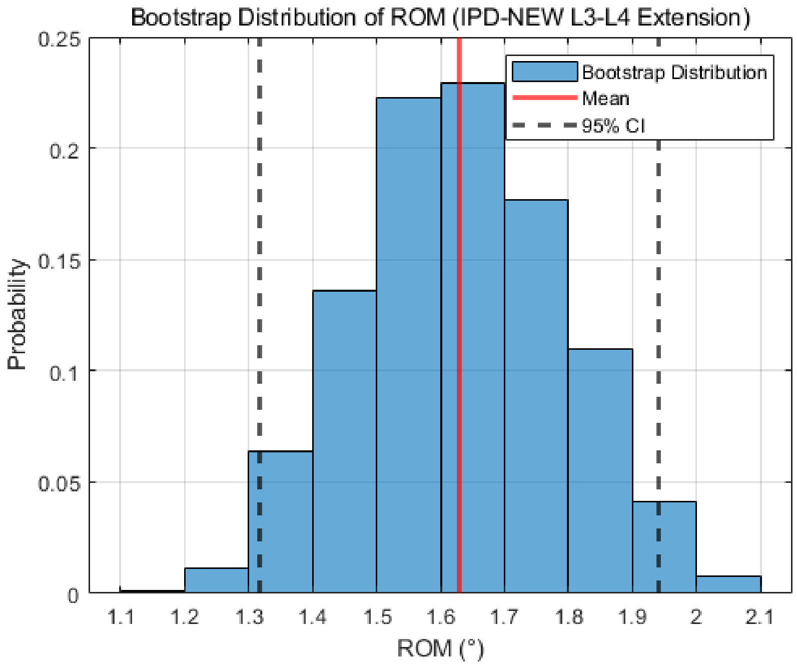

Supplementary Material Figure S8. Bootstrap Distribution of ROM (IPD-NEW L3-L4 Extension), 95% Confidence Interval: [1.32°, 1.94°]

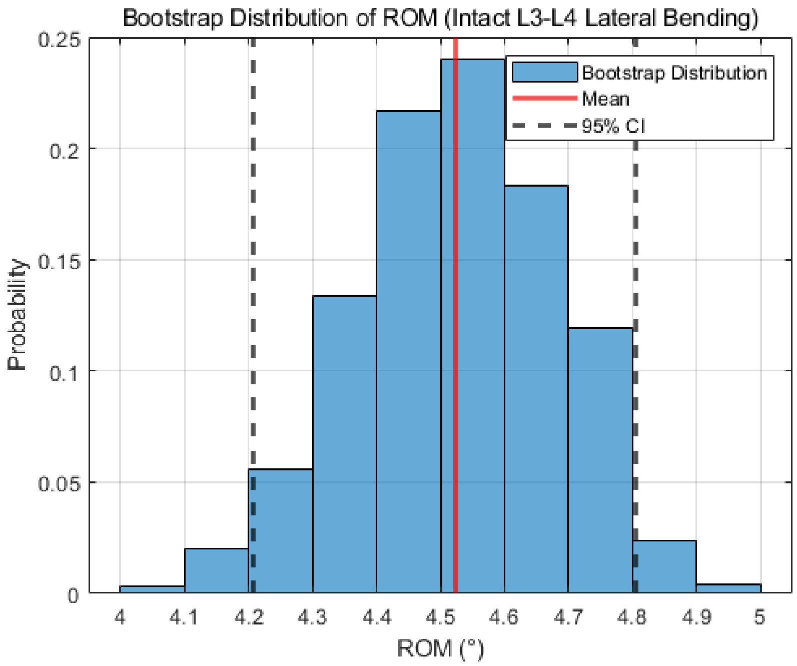

Supplementary Material Figure S9. Bootstrap Distribution of ROM (Intact L3-L4 Lateral Bending), 95% Confidence Interval: [4.21°, 4.81°]

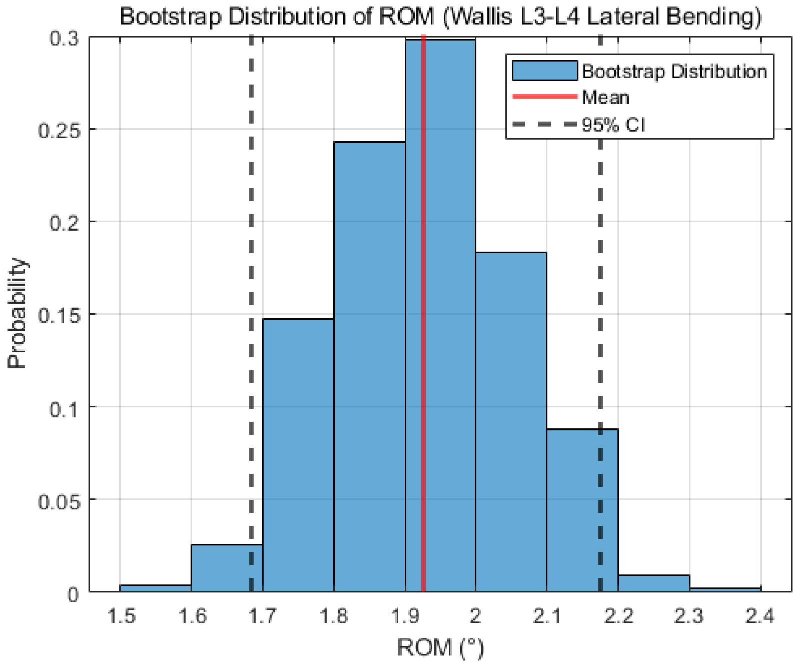

Supplementary Material Figure S10. Bootstrap Distribution of ROM (Wallis L3-L4 Lateral Bending), 95% Confidence Interval: [1.68°, 2.18°]

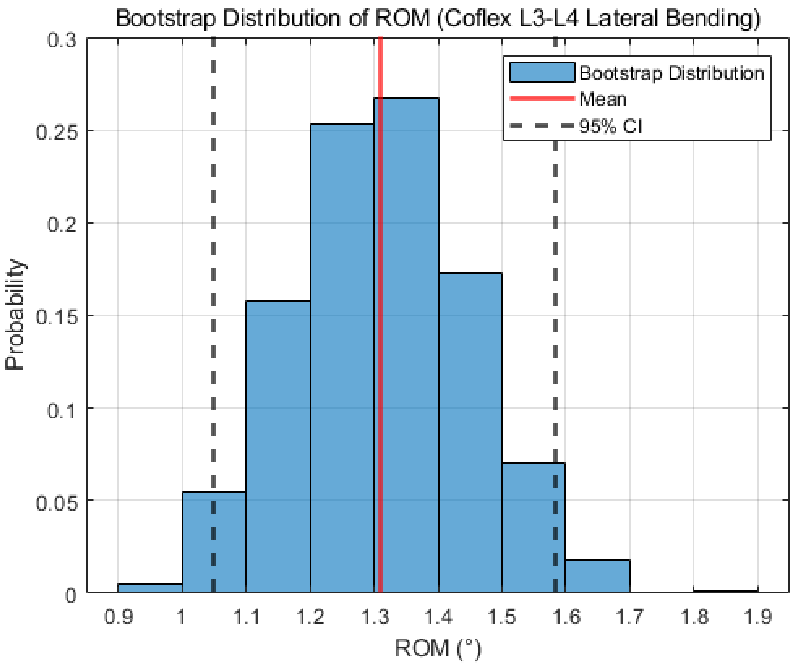

Supplementary Material Figure S11. Bootstrap Distribution of ROM (Coflex L3-L4 Lateral Bending), 95% Confidence Interval: [1.05°, 1.58°]

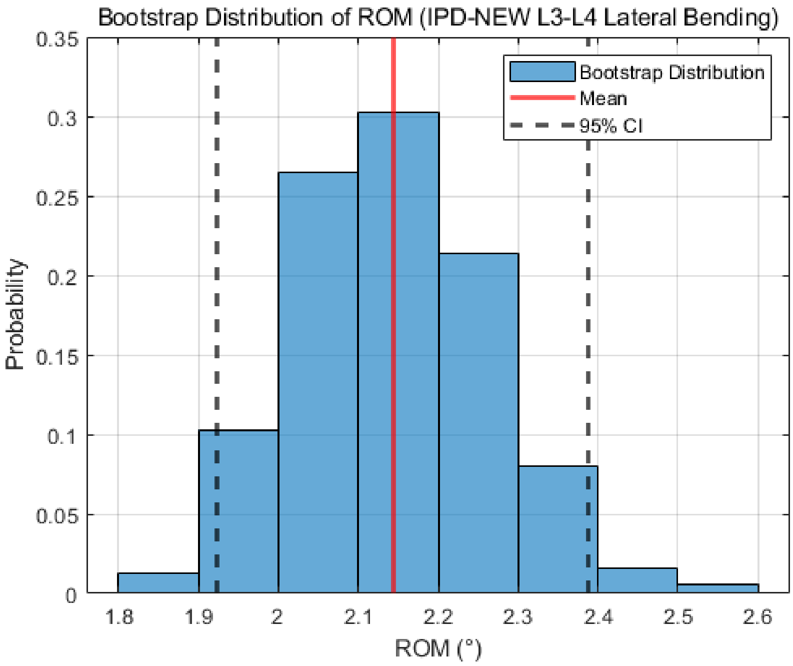

Supplementary Material Figure S12. Bootstrap Distribution of ROM (IPD-NEW L3-L4 Lateral Bending), 95% Confidence Interval: [1.92°, 2.39°]

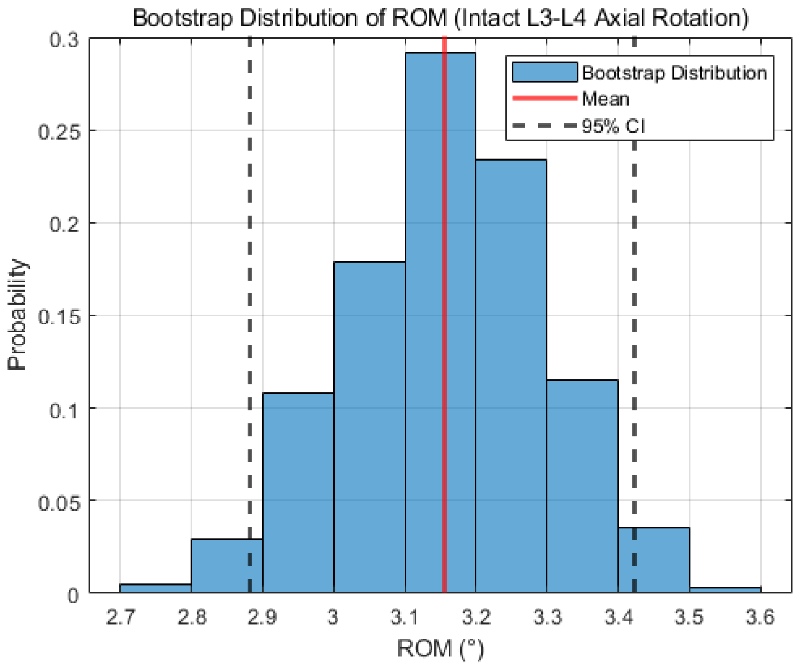

Supplementary Material Figure S13. Bootstrap Distribution of ROM (Intact L3-L4 Axial Rotation), 95% Confidence Interval: [2.88°, 3.42°]

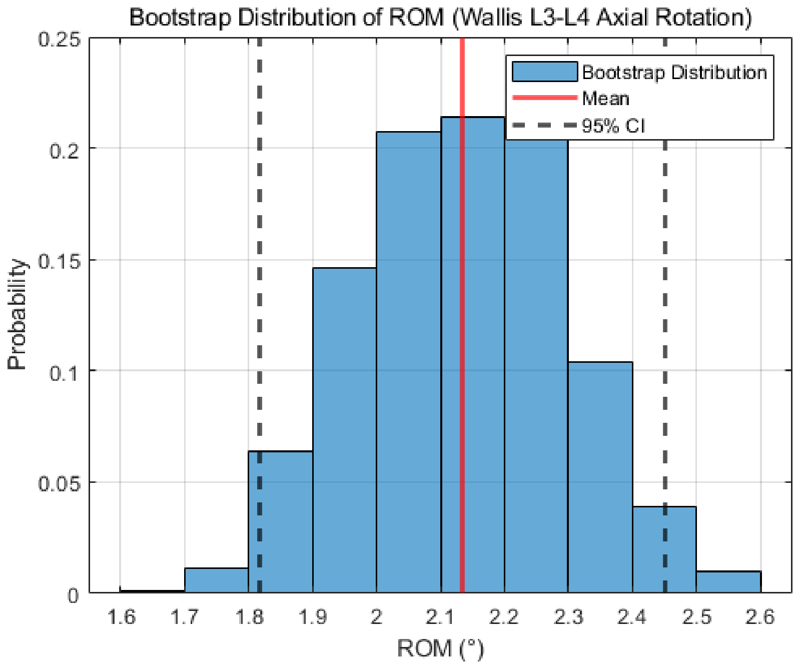

Supplementary Material Figure S14. Bootstrap Distribution of ROM (Wallis L3-L4 Axial Rotation), 95% Confidence Interval: [1.82°, 2.45°]

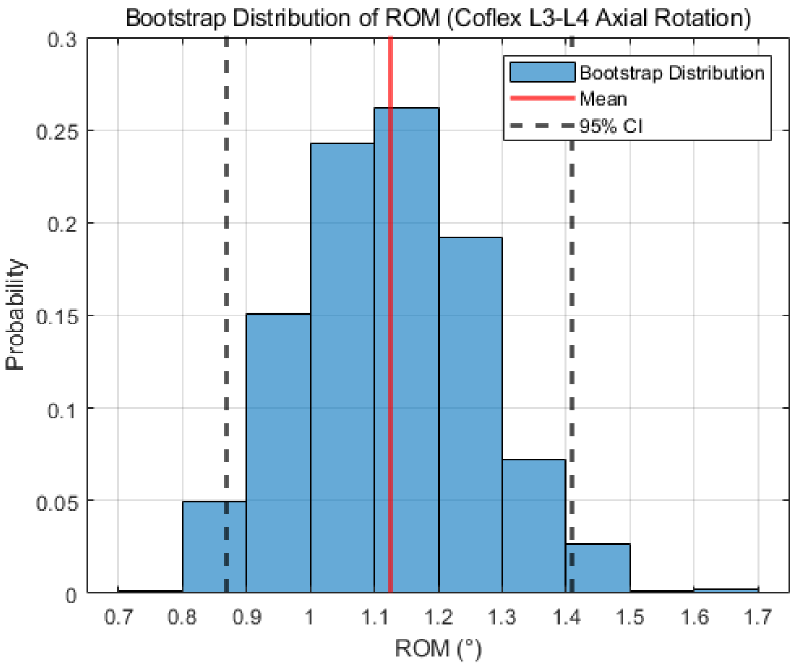

Supplementary Material Figure S15. Bootstrap Distribution of ROM (Coflex L3-L4 Axial Rotation), 95% Confidence Interval: [0.87°, 1.41°]

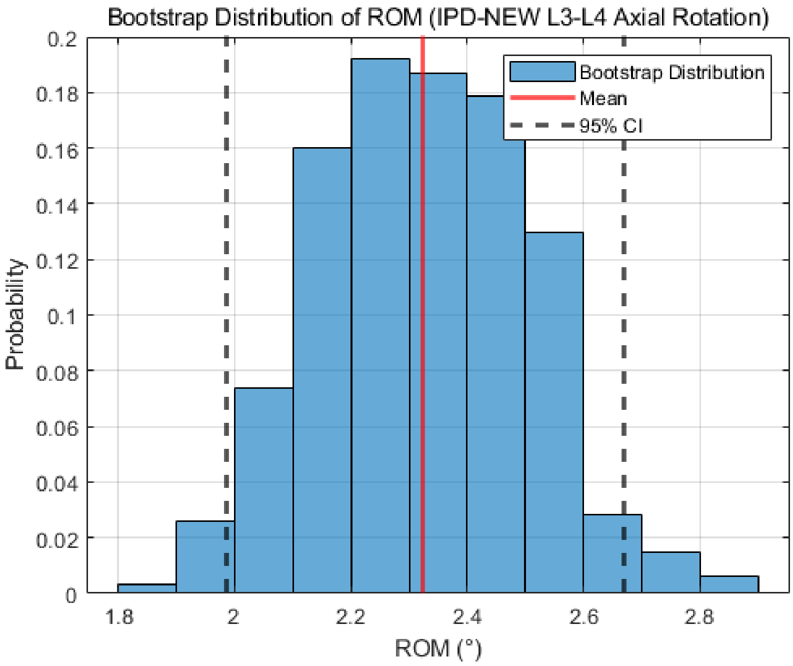

Supplementary Material Figure S16. Bootstrap Distribution of ROM (Coflex L3-L4 Axial Rotation), 95% Confidence Interval: [1.99°, 2.67°]
